# Factors Associated With the Development of Chronic Post-Sternotomy Pain: a Case-Control Study

**DOI:** 10.5935/1678-9741.20150059

**Published:** 2015

**Authors:** Mário Augusto Cray da Costa, Conrado Auer Trentini, Marcelo Derbli Schafranski, Oswaldo Pipino, Ricardo Zanetti Gomes, Elise Souza dos Santos Reis

**Affiliations:** 1 Universidade Estadual de Ponta Grossa (UEPG), Ponta Grossa, PR, Brazil, and Santa Casa de Misericórdia de Ponta Grossa, Ponta Grossa, PR, Brazil.; 2 Universidade Estadual de Ponta Grossa (UEPG), Ponta Grossa, PR, Brazil.; 3 Santa Casa de Misericórdia de Ponta Grossa, Pronta Gross, PR, Brazil.

**Keywords:** Chronic Pain, Cardiac Surgical Procedures, Sternotomy

## Abstract

**OBJECTIVE:**

The aim of the present study was to investigate the factors associated with chronic post-sternotomy pain in heart surgery patients.

**METHODS:**

Between January 2013 and February 2014, we evaluated 453 patients with >6 months post-sternotomy for cardiac surgery at a surgical outpatient clinic. The patients were allocated into a group with chronic post-sternotomy pain (n=178) and a control group without pain (n=275). The groups were compared for potential predictors of chronic post-sternotomy pain. We used Cox proportional hazards regression to determine which independent variables were associated with the development of chronic post-sternotomy pain.

**RESULTS:**

In total, 39.29% of the patients had chronic poststernotomy pain. The following factors were significantly associated with chronic post-sternotomy pain: (a) use of the internal thoracic artery in coronary bypass grafting (*P*=0.009; HR=1.39; 95% CI, 1.08 to 1.80); (b) a history of antidepressant use (*P*=0.0001; HR=2.40; 95% CI, 1.74 to 3.32); (c) hypothyroidism (*P*=0.01; HR=1.27; 95% CI, 1.03 to 1.56); (d) surgical wound complication (*P*=0.01; HR=1.69; 95% CI, 1.08 to 2.63), and (e) patients on disability benefits or scheduled for a consultative medical examination for retirement (*P*=0.0002; HR=2.05; 95% CI, 1.40 to 3.02).

**CONCLUSION:**

The factors associated with chronic poststernotomy pain were: use of the internal thoracic artery; use of antidepressants; hypothyroidism; surgical wound complication, and patients on disability benefits or scheduled for a consultative examination.

**Table:** 

**Abbreviations, acronyms & symbols**	
CI	= Confidence interval
COPD	= Chronic obstructive pulmonary disease
CPOP	= Chronic postoperative pain
CPSP	= Chronic post-sternotomy pain
HR	= Hazard ratio
ITA	= Internal thoracic artery
OR	= Odds ratio
SAH	= Systemic arterial hypertension

## INTRODUCTION

Chronic postoperative pain (CPOP) is defined as pain of onset following a surgical procedure and persisting for more than 2 months without other apparent causes such as infection or the underlying disease that motivated the surgery^[1]^.

The concept of CPOP was first proposed by Crombie, in 1998^[2]^; thereafter, various studies have been performed in an attempt to identify, define, quantify, and stratify the associated factors leading to CPOP. Median sternotomy is the most widely used approach in cardiac surgery and, despite all the advances in this field, a large number of patients report postoperative chest pain. The incidence of chronic post-sternotomy pain (CPSP) varies between 17% and 56%^[3]^-^[5]^; in approximately one-third of these patients, CPSP can compromise quality of life, affecting their sleep patterns and impairing their working ability^[3]^.

In addition to the diverse characteristics of pain in the various body tissues, sensitivity to pain varies between individuals depending on age, gender, and the underlying disease itself^[5]^. Other psychosocial factors such as depression, anxiety, low education level and fear of surgery are established risk factors for CPOP^[6]^,^[7]^.

Even though CPOP is frequently regarded as expected or irrelevant, it should be seen as a major clinical - and even social - issue. Chronic pain is often responsible for delayed return to work, which entails greater burden on the public health system and social security.

The aim of the present study was to determine some factors associated with CPSP in patients who underwent cardiac surgery at the Santa Casa de Misericórdia Hospital in Ponta Grossa, Paraná, Brazil.

## METHODS

After approval by the Human Research Ethics Committee of the Universidade Estadual de Ponta Grossa (Ponta Grossa State University) under Formal Opinion 159.531, we performed an observational analytical case-control study to examine the influence of a number of factors on the development of CPSP following heart surgery.

### Patients

We evaluated 453 consecutive patients with more than 6 months post-sternotomy for cardiac surgery assisted at the Heart Surgery and Cardiology Outpatient Clinic and private practice office of the Heart Surgery Service of the Santa Casa de Misericórdia Hospital of Ponta Grossa in the years 2013 through 2014. The time from surgery to evaluation ranged from 6 to 279 months, mean 58 months. All of these patients agreed to complete a pain questionnaire. Clinical and surgical data were complemented with data from the patients' medical records. Patients were included if they had undergone valve surgery, coronary artery bypass surgery, interatrial communication closure, aortic surgery, left ventricle aneurysm repair, myxoma resection or combined procedures in which sternotomy was the selected approach to the thoracic cavity.

### Exclusion criteria

Patients with less than 6 months of surgery and patients with connective tissue disorders were excluded. There weren't other exclusion criteria.

### Study Variables

The patients were inquired with respect to the presence of chest pain, its characteristics, and intensity ranging from 1 to 10. For the aim of our study, only the binary variable "presence or absence of pain" was taken into account. Chronic pain was defined as new-onset pain that arose postoperatively, persisting for more than 6 months after the surgery. Macrae^[1]^ defined CPOP as pain originated after a surgical procedure and lasting at least 2 months. In the present study, as a factor of safety, we only included patients with a postoperative period > 6 months. Any sternal or anterior chest pain, regardless of its intensity, was taken into account and provided that the pain was present at the time of evaluation.

The patients were allocated into a group with chronic pain and a control group without pain (dependent variable). The following independent variables were included in the Cox regression model: (a) age; (b) gender; (c) foreign descent (patients whose parents were not born in Brazil. There are a lot of European descents in the study and it was intended to assess whether this population has different sensitivity to pain); (d) use of internal thoracic artery (ITA) grafts in coronary artery bypass surgery; (e) depression or anxiety that needed use of antidepressant medication at any point in time for treatment to exclude any minor episode of depression or anxiety that did not need use of medications; (f) diabetes mellitus (only patients who were in treatment for diabetes at the time of evaluation); (g) chronic obstructive pulmonary disease (COPD) clinically confirmed by patient history, physical examination, and use of COPD medication; (h) systemic arterial hypertension (SAH) (only patients in antihypertensive treatment at the time of evaluation; (i) hypothyroidism (patients with confirmed disease by TSH level and on thyroid hormone replacement therapy); (j) dyslipidemia (patients with history of dyslipidemia and using lipid-lowering agents); (k) surgical wound complication (resolved superficial wound infection, cured mediastinitis, keloid formation, or dehiscence of healed wound), and (l) patient on disability benefits or scheduled for a consultative medical examination for retirement purposes.

The clinical criteria were based on history, laboratorial exams and disease treatment, since the patients were receiving optimized medical treatment and in the late postoperative period with multiple medications.

### Statistical Analysis

The data were analyzed using the Cox proportional hazards model. The Cox model makes it possible to determine which independent variables have an intensifying effect when analyzed as a set, and computes their statistical significance within a confidence interval (CI) 95%. Thus, we were able to estimate the hazard ratio (HR) of each of these variables. Values of *P*<0.05 were considered statistically significant.

## RESULTS

We evaluated 453 patients; 178 (39.29%) of these had CPSP (Table 1). The prevalence of factors related to the event of CPSP was compared between the groups and is shown in Table 2.

**Table 1:** Pain stratification by intensity from 0 to 10.

Intensity	Patients	%
No pain	275	60.7
1 and 2	62	13.7
3 and 4	32	7.1
5 and 6	56	12.4
7 and 8	16	3.5
9 and 10	12	2.6

**Table 2:** Factors associated with the development of CPSP^[a]^.

Patient's demographics	With Pain n=178 (39.29)	Without Pain n=275 (60.7)
**Age (years)**		
Mean (SD)	58 (10.9)	62 (12.6)
**Gender**		
Male	94 (52.8)	165 (60)
Female	84 (47.2)	110 (40)
**Work status**		
Retired	92 (51.6)	181 (65.8)
Disability benefits/Scheduled for consultative medical examination	43 (24.1)	30 (10.9)
Working	13 (7.3)	21 (7.6)
Unemployed	4 (2.2)	7 (2.5)
**Self-employed**	26 (14.6)	36 (13.1)
**Foreign descent**	53 (19.1)	100 (36.4)
**Depression/anxiety**	116 (65.2)	79 (28.7)
**Comorbidities**		
Diabetes mellitus	48 (26.9)	58 (21.1)
COPD	16 (8.9)	41 (14.9)
SAH	148 (83.1)	217 (78.9)
Dyslipidemia	125 (70.2)	177 (64.3)
Hypothyroidism	29 (16.3)	34 (12.3)
**Use of mammary graft**	120 (67.4)	138 (50.2)
**Wound complication^b^**	25 (14)	7 (2.5)
**Time between surgery and evaluation (months)**	46 (25.8)	65 (23.6)

COPD=chronic obstructive pulmonary disease, SAH=systemic arterial hypertension

a Results presented as n (%)

b Infection, mediastinitis, keloid formation, or wound dehiscence

The mean age of CPSP patients was 58±10.9 years *vs.* 62±12.6 years for the control patients. In all, 94 (52.8 %) patients were male and 84 (47.2%), female.

Of the patients who developed CPSP, 120 (67.4%) received internal mammary artery grafts; 116 (65.2%) patients had depression or anxiety disorders at some point in their lives; 29 (16.3%) had hypothyroidism, and 25 (14%) underwent postoperative sternotomy wound complications. With regard to work status, 92 (51.6%) patients were retired; 43 (24.1%) were receiving disability benefits or scheduled for a consultative medical exam; 13 (7.3%) were working; 4 (2.2%) patients were unemployed, and 26 (14.6%) were self-employed.

The results of the statistical analysis by Cox regression are presented in Table 3.

**Table 3:** Statistical analysis using Cox's regression model

Variable	Hazard Ratio	95% CI	P-value
Age	0.98	0.97 - 1.00	0.17
Male	1.12	0.82 - 1.53	0.46
Foreign descent	0.77	0.55 - 1.08	0.14
Use of mammary artery graft	1.39	1.08 - 1.80	0.009
Depression or anxiety	2.40	1.74 - 3.32	0.0001
Diabetes mellitus	0.85	0.59 - 1.23	0.40
COPD	0.95	0.64 - 1.40	0.81
SAH	1.18	0.74 - 1.88	0.48
Hypothyroidism	1.27	1.03 - 1.56	0.01
Dyslipidemia	0.84	0.54 - 1.32	0.46
Wound complication^a^	1.69	1.08 - 2.63	0.01
Disability benefits/Scheduled for consultative medical examination	2.05	1.40 - 3.02	0.0002

COPD=chronic obstructive pulmonary disease; SAH=systemic arterial hypertension

a Infection, mediastinitis, keloid formation, or wound dehiscence

The following variables were statistically significant according to Cox model of logistic regression: ITA grafts (*P*=0.009; HR=1.39; 95% CI, 1.08 to 1.80); history of use of depression or anxiety medication (P=0.0001; HR=2.40; 95% CI, 1.74 to 3.32); hypothyroidism (P=0.01; HR=1.27; 95% CI, 1.03 to 1.56); surgical wound complication (P=0.01; HR=1.69; 95% CI, 1.08 to 2.63), and patients on disability benefits or scheduled for a consultative medical examination for retirement (P=0.0002; HR=2.05; 95% CI, 1.40 to 3.02).

## DISCUSSION

According to the literature, the incidence of CPSP ranges from 17% to 56%^[2]^-^[5]^. This is consonant with the results of the present sample, which showed an incidence rate of 39.29%.

An extensive review of the literature allowed us to conclude that the present study involved the world's largest number of published cases of patients interviewed face-to-face in CPSP research.

In our study, 67.4% of the patients with CPSP had received an ITA graft. Mailis et al.^[8]^ described the association between postoperative thoracic pain and the use of ITA grafts in myocardial revascularization and indicated the possibility of a pain syndrome resulting from the use of that artery. This finding prompted studies aimed at proving the source of the pain. Eng & Wells^[9]^ reported that the use of ITA grafts resulted in a high incidence of CPSP compared with venous grafts in revascularization surgeries. Thus, the use of ITA coronary grafts emerged as one of the risk factors for chronic pain. This finding corroborates those of Alston & Pechon^[10]^, who affirmed that damage to the intercostal nerves could occur in the dissection of the ITA bed using diathermy.

Intraoperative nerve injury is a major cause of pain manifest postoperatively as hyperalgesia and allodynia, both typical symptoms of neuropathic pain^[11]^. Van Gulik et al.^[12]^, in a prospective cohort study with 146 patients who underwent heart surgery by sternotomy, found that 54.8% of the patients with CPSP had received ITA revascularization (OR=1.27; 95% CI, 0.60-2.70), values that are close to those of our study (67.4 %; HR=1.39; 95% CI, 1.08-1.80). Because OR and HR are effect size measures, both resulting from a regression study, OR and HR values are comparable.

The proportional hazards regression of Cox made it possible to demonstrate the relationship between CPSP and depression or anxiety disorder. Romano & Turner^[13]^ performed a study that provided evidence of the association of chronic pain with depression. Ohayon & Schatzberg^[14]^, in a 2010 study with 3243 patients, informed that 66.3% of the patients with depression reported chronic pain in the most diverse locations. In the present study, 65.2% of the patients with CPSP had depression or anxiety disorder. If analyzed separately, 59.4% among the patients diagnosed with depression or anxiety disorder had chronic pain, which is close to the percentage found in the Ohayon & Schatzberg^[14]^ study. Nevertheless, data are still lacking in the literature correlating CPSP to depression or anxiety disorder.

The origin of this type of pain can be explained by a number of theories, with depression or anxiety disorder and pain being related on the most diverse levels, such as the psychological, behavioral, and neurobiological. Neurotransmitters such as serotonin and noradrenaline, both implicated in depressive or anxiety disorders, act on the neurobiological level and play an important role in the modulation of pain. Osterweis et al.^[15]^ stated that, on account of the action of neurotransmitters, pain conduction can in fact be altered by depression in the same way as a nociceptive impulse is capable of inducing or exacerbating a given mood. It has been hypothesized that, psychologically, chronic pain is a particular form of somatization in which negative emotions are expressed through physical complaints - including pain. Somatosensory amplification is a related concept that has been defined as a person's increased propensity to feel and express pain. Based on the explanation for these complex mechanisms, Von Korff & Simon.^[16]^ were able to prove the relationship between pain and depression, which was also found in the present study sample.

About dyslipidemia, all of the patients were using statin. Although statin can cause muscle pain there wasn't statistical differences in this variable. Hypothyroidism was also more common in the group of patients with CPSP. Many reports can be found in the literature concerning thyroid hormones and their functions; however, studies of their connection with pain are scarce. The thyroid gland has a prominent role in tissue metabolism and development; in addition, the hypothalamic-pituitary-thyroid axis is central to the modulation and neurotransmission processes in the central nervous system^[17]^.

Aloisi et al.^[17]^, in a pilot study with a group of 200 women with chronic pain - whose medical history was investigated - found that 19% of them had some degree of thyroid dysfunction. That rate is similar to the overall value of 16.3% found in our analysis.

Despite the number of existing studies, the hypothesized relationship between hypothyroidism and chronic pain is still far from understood. The origin of pain could be neuropathic; this is one of the key points to be elucidated, as neuropathic pain is prominent in somatosensory system injury^[18]^. Aminoglycan deposits surrounding nerves, primary axonal degeneration, and segmental demyelination mechanisms have been proposed for the peripheral nerve dysfunction in hypothyroidism, as demonstrated in rodents^[19]^. Another piece of evidence is the low oxygen consumption and high blood pressure frequently found among hypothyroid individuals, leading to regional hypoxia and eventually to muscle spasms due to the inadequate supply of blood and nutrients for the muscles^[20]^. This could explain some of the mechanisms involving hypothyroidism and pain.

As evidenced by Cox regression, sternal surgical wound complications are associated with the outcome under study. Sternal wound complications increase the risk of CPSP, ranging from superficial infections to sternal instability and mediastinitis^[21]^. Marques et al.^[22]^ pointed out that although wound complications following sternotomy are rare, infection is a threatening occurrence because it could extend to other areas, thus causing future complications.

Einsenberg et al.^[4]^ suggested that sternal surgical wound infection is one of the likely causes of CPSP. This had already been indicated by Ottino et al.^[23]^ in 1987. Although there are reports on the relationship between surgical wound complications and chronic pain, concrete data to evidence this association were still lacking.

Another risk factor for CPSP was identified in the patients who were receiving disability benefits or were scheduled for a consultative medical examination to apply for early retirement by the National Social Security Institute. Although no reports can be found in the literature addressing this specific variable and its relationship to CPSP, similar studies have been developed. In 1995, Rosomoff et al.^[24]^ studied the relationship between the perception of chronic pain by patients who derived some form of secondary gain from their complaint of pain and those who did not have such gain. Even though those authors suggested that there could be a link between altered perception of pain and the benefits obtained, they also stated that they were not able to demonstrate a difference between the group receiving benefits and the group without benefits.

Along these lines, Vaccaro et al.^[25]^, in a 1997 retrospective study with 24 patients who had chronic low back pain, found that 11 out of the 13 patients who had applied for worker's compensation showed poor outcomes in the management of their chronic pain while 9 of the 11 patients in the group of non-claimants had excellent results. Those authors concluded that although the sample size was small and the study was retrospective, worker's compensation and litigation claims were strongly associated with worse outcomes in the management of chronic pain.

Our clinical experience shows that the patients who have not yet retired and are not working complain of chronic pain more often, which was evidenced in the present case population. Psychological and behavioral mechanisms may be implicated in the enhanced pain of patients who expect to be granted social security benefits or who fear losing those benefits, since these beneficiaries have to undergo frequent consultative medical exams for years. On the other hand, given that patients with pain are more likely to seek disability benefits, CPSP may be either the cause or the effect in the case of patients on disability benefits.

## CONCLUSION

In the case patients of the present study, the factors associated with increased incidence of chronic post-sternotomy pain were: (a) use of ITA grafts; (b) history of depression or anxiety that needed use of antidepressant medication; (c) hypothyroidism; (d) surgical wound complications, and (e) patients on disability benefits or scheduled for a consultative examination.

**Table:** 

**Authors' roles & responsibilities**	
MACC	Conception and design; final approval of the manuscript
CAT	Analysis/interpretation of data; implementation of projects/experiments; manuscript writing/critical review of its content; statistical analysis; final approval of the manuscript
MDS	Study design; manuscript writing/ critical review of its content; final approval of the manuscript
OP	Conception and design; implementation of projects/ experiments; final approval of the manuscript
RZG	Analysis/interpretation of data; final approval of the manuscript
ESSR	Analysis/interpretation of data; implementation of projects/experiments; manuscript writing/critical review of its content; final approval of the manuscript
